# Trends of overweight and obesity prevalence in school-aged children among Henan Province from 2000 to 2019

**DOI:** 10.3389/fpubh.2022.1046026

**Published:** 2022-12-05

**Authors:** Yuhao Zhang, Hao Lou, Ye Huang, Ruijuan Wang, Xiao Wen, Cuiping Wu, Changfu Hao, Ran Li, Genli Gao, Xiaomin Lou, Xian Wang

**Affiliations:** ^1^College of Public Health, Zhengzhou University, Zhengzhou, Henan, China; ^2^Department of Nosocomial Infection Management, The First Affiliated Hospital of Zhengzhou University, Zhengzhou, Henan, China; ^3^Zhengzhou Hospital of Traditional Chinese Medicine, Zhengzhou, Henan, China; ^4^Zhengzhou Station for Students' Health, Zhengzhou, Henan, China; ^5^The Education Department of Henan Province, Zhengzhou, Henan, China

**Keywords:** overweight, obesity, trend, children and adolescents, prevalence

## Abstract

**Objectives:**

Overweight and obesity are harmful to human health. However, the latest trends of Chinese childhood overweight and obesity prevalence are not available. The aim of this study was to examine the trends from 2000 to 2019 among students in China.

**Methods:**

We analyzed data of 66,072 students in the Chinese National Survey on Students' Constitution and Health from 2000 to 2019. Overweight and obesity were defined based on the standard formulated by the International Obesity Task Force (IOTF standard), the World Health Organization (WHO standard), and the Working Group on Obesity in China (WGOC standard), respectively. The χ^2^-test was used to test the trends of overweight and obesity prevalence and logistic regression was conducted to evaluate the prevalence odds ratios of boys vs. girls and urban vs. rural areas.

**Results:**

The prevalence of obesity/overweight and obesity combined was 6.03/23.58% (IOTF standard), 10.56/25.88% (WGOC standard) and 10.75/29.69% (WHO standard) in 2019. From 2000 to 2019, according to the WGOC standard, the prevalence increased from 2.51 to 10.56% for obesity and increased from 9.81 to 25.88% for overweight and obesity combined (*P* for trend < 0.001). Obesity/overweight and obesity were greater problems in boys than girls and urban than rural areas, but urban-rural differences decreased over time.

**Conclusion:**

Overweight and obesity prevalence increased significantly in children and adolescents in China from 2000 to 2019. The prevalence of overweight and obesity in rural areas may contribute to a large percentage of children with overweight and obesity.

## Introduction

The epidemic of childhood overweight and obesity is now considered to be a major global public health problem in the 21st century (1, 2). Globally, the age-standardized prevalence of obesity increased from 0.7 to 5.6% for girls and from 0.9 to 7.8% for boys among children aged 5–19 years from 1975 to 2016 (3). Although the growth rate of childhood obesity prevalence had slowed down, evidence showed that childhood overweight prevalence was still on the rise from 2009 to 2019 in China (4).

The effect of overweight and obesity on hypertension, type 2 diabetes, and other cardiovascular diseases (CVD) among children and adults have been reported previously (5–8). A recent cohort study indicated that overweight or obese children were more likely to have alanine aminotransferase (ALT) elevation and non-alcoholic fatty liver disease (NAFLD) in adulthood (9). Furthermore, children obesity was found to increase the risk of lifetime major depressive disorder (MDD) (10). Several recent studies revealed that overweight and obesity were also harmful factors of coronavirus disease 2019 (COVID-19) (11, 12).

There are region and socioeconomic disparities in overweight and obesity prevalence (13). The economic level of Henan province has grown rapidly in the past two decades. With the economic growth, great changes have taken place in people's lifestyle. In parallel to the changes, the rate of overweight and obesity may be increased (14). In addition, Henan province is a province with a large population in central of China, and there are a large number of primary and secondary school students. In view of the scarcity of research in localized area, research is urgently needed to identify trends of overweight and obesity among school-aged children in Henan province.

This study used the data from the Chinese National Survey on Students' Constitution and Health (CNSSCH) of Henan province to assess the latest overweight and obesity prevalence in 2019 and to evaluate the secular trends of overweight and obesity prevalence from 2000 to 2019 among children and adolescents in Henan province so as to provide latest information for policymakers.

## Subjects and methods

### Subjects

Data were acquired from 2000, 2005, 2010, 2014, and 2019 cycles of the CNSSCH of Henan province, which was the most representative survey about the physical condition of Chinese school-aged children (15, 16). According to the socioeconomic status (SES), upper, middle and lower socioeconomic region in Henan province were selected, corresponding to Zhengzhou city, Xinxiang city and Zhoukou city. And then, one urban survey region and one rural survey region were selected from each city. The participants were selected from the same survey region in Henan province from 2000 to 2019. More than 85% of the sampled schools remained the same in all surveys (17). The subjects aged 7–18 years in school were chosen by stratified cluster sampling. The ratio of boys to girls and urban students to rural students approximately equaled 1:1 in each survey (Table 1). The participants were classified as four groups: urban boys, rural boys, urban girls and rural girls. Besides, the number of participants in each group from upper, middle and lower socioeconomic regions was nearly the same. The inclusion criteria were that the participants were in good physical condition and could engage in various physical exercise activities. The participants were excluded from the research if they had one of the following conditions: (1) serious organ diseases; (2) abnormal physical or deformity; (3) acute diseases such as fever, diarrhea. The inclusion and exclusion criteria were consistent in each survey. Finally, a total of 14 081 school aged children in 2000, 8 639 in 2005, 14 395 in 2010, 14 423 in 2014 and 14 534 in 2019, with integral data were included in the analysis (Table 1). The research protocol was approved by the Zhengzhou University Life Science Ethics Committee (ZZUIRB2021-56). Informed consent of parents and their children were obtained.

**Table 1:** The sample size for different survey years of the CNSSCH in Henan province, China.

**Years *n* (%)**	**Total**	**Location** ***n*** **(%)**		**Sex** ***n*** **(%)**		**Age group** ***n*** **(%)**			
		**Urban areas**	**Rural areas**	**Boys**	**Girls**	**7–9 y**	**10–12 y**	**13–15 y**	**16–18 y**
2000	14,081	7,089 (50.34)	6,992 (49.66)	7,052 (50.08)	7,029 (49.92)	3,598 (25.55)	3,600 (25.57)	3,598 (25.55)	3,285 (23.33)
2005	8,639	4,320 (50.01)	4,319 (49.99)	4,320 (50.01)	4,319 (49.99)	2,160 (25.00)	2,160 (25.00)	2,160 (25.00)	2,159 (25.00)
2010	14,395	7,198 (50.00)	7,197 (50.00)	7,196 (49.99)	7,199 (50.01)	3,599 (25.00)	3,599 (25.00)	3,597 (24.99)	3,600 (25.01)
2014	14,423	7,207 (49.97)	7,216 (50.03)	7,211 (50.00)	7,212 (50.00)	3,603 (24.98)	3,606 (25.00)	3,606 (25.00)	3,608 (25.02)
2019	14,534	7,343 (50.52)	7,191 (49.48)	7,256 (49.92)	7,278 (50.08)	3,688 (25.38)	3,615 (24.87)	3,596 (24.74)	3,635 (25.01)
χ^2^-value		1.38		0.07		17.84			
*P*-value		0.848		0.999		0.121			

### Measurements

Height and weight in all surveys were measured according to standardized procedures as previously reported (18). All the measurements were implemented by professionals who had passed correlative training courses. Values of body mass index (BMI) were calculated (5).

### Definition

BMI was used to judge overweight or obesity based on the standard defined by Working Group on Obesity in China (WGOC) (19). Those with BMI values 85th centile or above but < 95th centile were classified as overweight children, and those with BMI values 95th centile or above were classified as obese children. The cut-offs of BMI for 18 years old group were defined to be 24 for overweight and 28 for obesity (20). Based on the population in 2000 CNSSCH data, the WGOC standard was suitable for the Chinese school-aged children (19). Besides, in order to compare with domestic and foreign research results, the International Obesity Task Force (IOTF) standard and World Health Organization (WHO) standard were also used to distinguish overweight and obesity (21, 22). In our study, we used the above three standards to evaluate overweight and obesity prevalence in 2019, and used WGOC standard to assess the trends of overweight and obesity prevalence from 2000 to 2019.

### Statistical analysis

Results were described as mean ± standard deviation (SD) for continuous variables, and percentages and frequencies for categorical ones. χ^2^ test was used to test the differences of distributions in locations, gender, and age groups between five surveys, the differences of overweight and obesity prevalence, and the trends of overweight and obesity prevalence across years in different subgroups. To acquire sex differences and urban-rural disparity in different survey years, the binary logistic regression was applied to evaluate the prevalence odds ratios (POR) and 95% confidence intervals (CI) of boys compared to girls and urban areas vs. rural areas with adjustment for region, sex, location and age. All analyses were performed using SPSS 21.0. Two-sided *P*-values < 0.05 were regarded to be statistically significant.

## Results

### Prevalence of overweight and obesity calculated by three standards in 2019

As shown in Table 2, the study participants were divided into different groups according to region, location and sex. Based on the IOTF, WGOC and WHO standards, obesity prevalence among children was 6.03, 10.56, and 10.75%, respectively, while the overweight and obesity combined prevalence was 23.58, 25.88, and 29.69%, respectively. In the regional subgroups, the prevalence of obesity and that of overweight and obesity combined were significantly different between regions according to each of three standards (all *P* < 0.001). Obesity prevalence according to each of three standards was highest in children among lower socioeconomic regions. The results of overweight and obesity combined presented the same epidemic characteristics. Based on the above three standards, the prevalence of obesity and that of overweight and obesity combined were dramatically different among different locations and sex (all *P* < 0.001). The participants in urban areas presented higher obesity/overweight and obesity combined rates than those in rural areas. The prevalence of obesity/overweight and obesity combined in boys were more than that in girls. The differences in prevalence among the three criteria were statistically significant in both the general population and subgroups (all *P* < 0.001).

**Table 2:** The prevalence of overweight and obesity determined by three standards in Henan Province, China in 2019.

**Classification**	**Obesity** ***n*** **(%)**			**Overweight and obesity** ***n*** **(%)**		
	**IOTF standard**	**WGOC standard**	**WHO standard**	**IOTF standard**	**WGOC standard**	**WHO standard**
All	876 (6.03)	1,535 (10.56)	1,563 (10.75)	3,427 (23.58)	3,762 (25.88)	4,315 (29.69)
Region						
Upper socioeconomic region	235 (4.88)	427 (8.86)	438 (9.09)	1,036 (21.49)	1,141 (23.67)	1,349 (27.99)
Middle socioeconomic region	214 (4.40)	394 (8.09)	419 (8.61)	960 (19.72)	1,051 (21.59)	1,225 (25.16)
Lower socioeconomic region	427 (8.81)	714 (14.73)	706 (14.57)	1,431 (29.53)	1,570 (32.40)	1,741 (35.93)
χ^2^-value	100.48	135.48	110.81	147.07	166.26	144.75
*P*-value	< 0.001	< 0.001	< 0.001	< 0.001	< 0.001	< 0.001
Location						
Urban areas	544 (7.41)	933 (12.71)	959 (13.06)	2,015 (27.44)	2,174 (29.61)	2,467 (33.60)
Rural areas	332 (4.62)	602 (8.37)	604 (8.40)	1,412 (19.64)	1,588 (22.08)	1,848 (25.70)
χ^2^-value	49.99	72.26	82.23	122.84	107.19	108.56
*P*-value	< 0.001	< 0.001	< 0.001	< 0.001	< 0.001	< 0.001
Sex						
Boys	624 (8.60)	965 (13.30)	1,126 (15.52)	2,070 (28.53)	2,252 (31.04)	2,619 (36.09)
Girls	252 (3.46)	570 (7.83)	437 (6.00)	1,357 (18.65)	1,510 (20.75)	1,696 (23.30)
χ^2^-value	169.31	114.99	342.66	196.95	200.50	284.79
*P*-value	< 0.001	< 0.001	< 0.001	< 0.001	< 0.001	< 0.001

WGOC, Working Group on Obesity in China; IOTF, International Obesity Task Force; WHO, World Health Organization.

### Trends in overweight and obesity prevalence from 2000 to 2019

**Figure 1:**
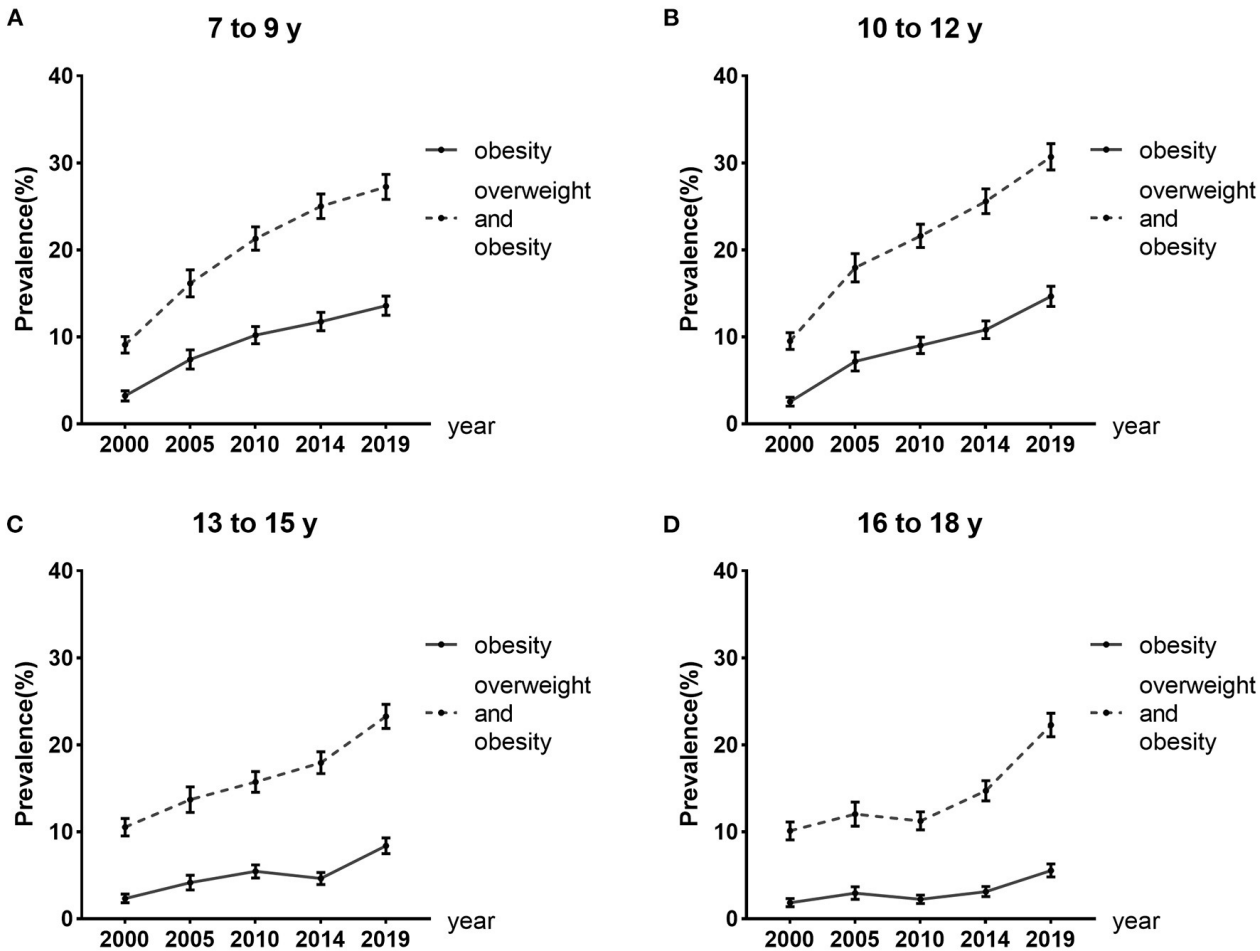
Trends in overweight and obesity prevalence from 2000 to 2019 in different age groups. Error bars represent 95% confidence intervals.

Figure 1 and Supplementary Table 1 showed that childhood obesity prevalence increased significantly over the past 20 years based on the WGOC standard (all *P* trend test < 0.001). The obesity rate increased from 2.51% (95%CI: 2.25–2.77%) to 10.56% (95%CI: 10.06–11.06%). After stratified by age, an increasing trend in the obesity prevalence was observed in all age groups from 2000 to 2019 (all *P* trend test < 0.001), and this increase was marked in the 10–12 years old subgroup, from 2.56% (95%CI: 2.04–3.07%) to 14.66% (95%CI: 13.51–15.81%). For the participants overall, overweight and obesity combined prevalence increased significantly across the five study periods (all *P* trend test < 0.001), from 9.81% (95%CI: 9.32–10.30%) in 2000 to 25.88% (95%CI: 25.17–26.60%) in 2019. The increasing trends were also observed in each age group (all *P* trend test < 0.001). The dramatic increment also occurred in 10 to 12 years from 9.53% (95%CI: 8.57–10.49%) to 30.71% (95%CI: 29.20–32.21%).

**Figure 2:**
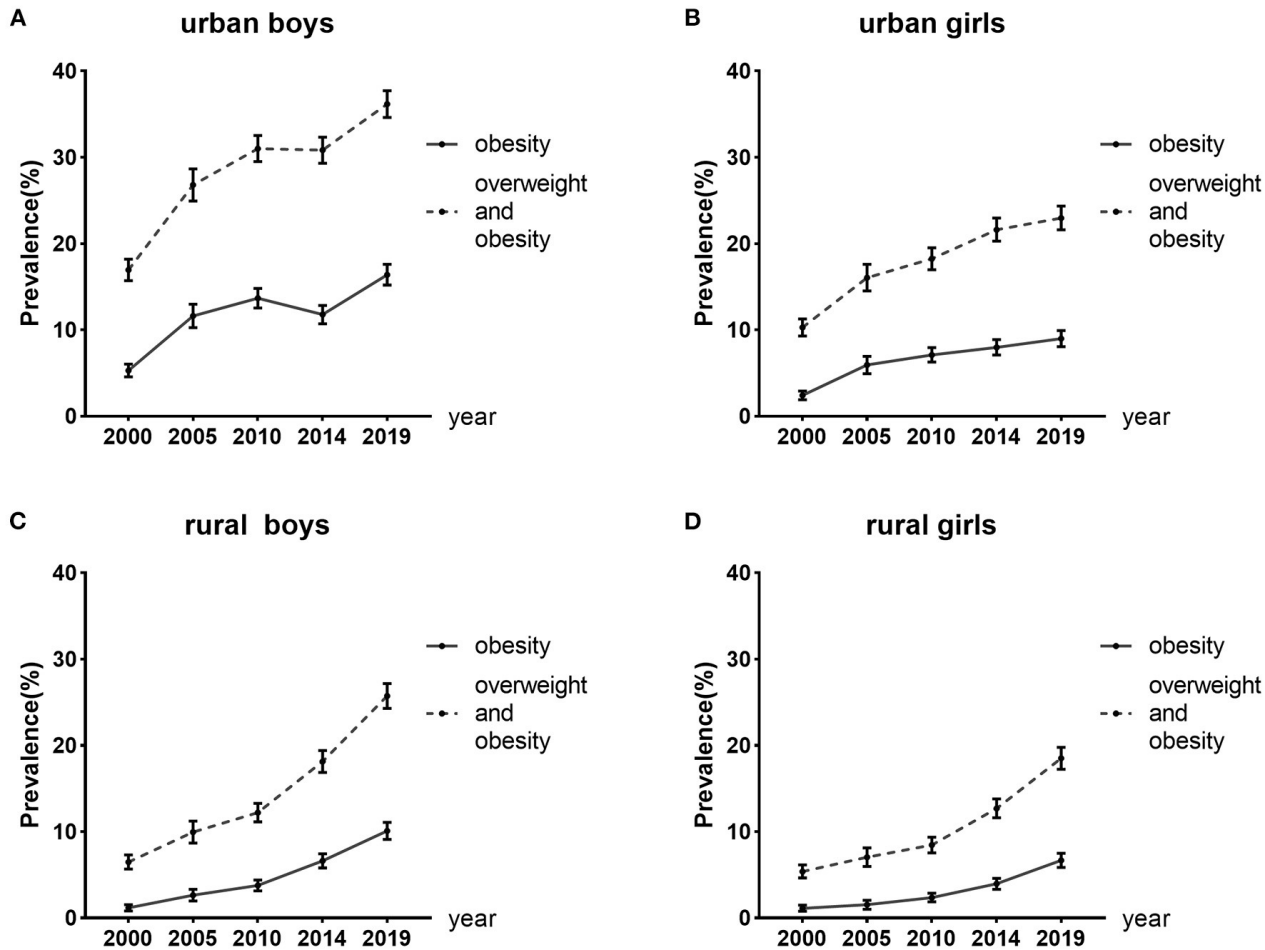
Trends in overweight and obesity prevalence from 2000 to 2019 in different location and sex. Error bars represent 95% confidence intervals.

After stratified by location and sex, the increasing trend in the obesity prevalence was observed in all subgroups from 2000 to 2019 (all *P* trend test < 0.001), and this increase was prominent in the urban boys, from 5.29% (95%CI: 4.56–6.03%) to 16.39% (95%CI: 15.20–17.59%). The prevalence of overweight and obesity combined also increased significantly in all subgroups (all *P* trend test < 0.001) and an obvious increase was observed in rural boys from 6.49% (95%CI: 5.67–7.30%) to 25.72% (95%CI: 24.29–27.16%) (Figure 2 and Supplementary Table 2).

### PORs of boys vs. girls and urban vs. rural children for overweight and obesity

**Figure 3:**
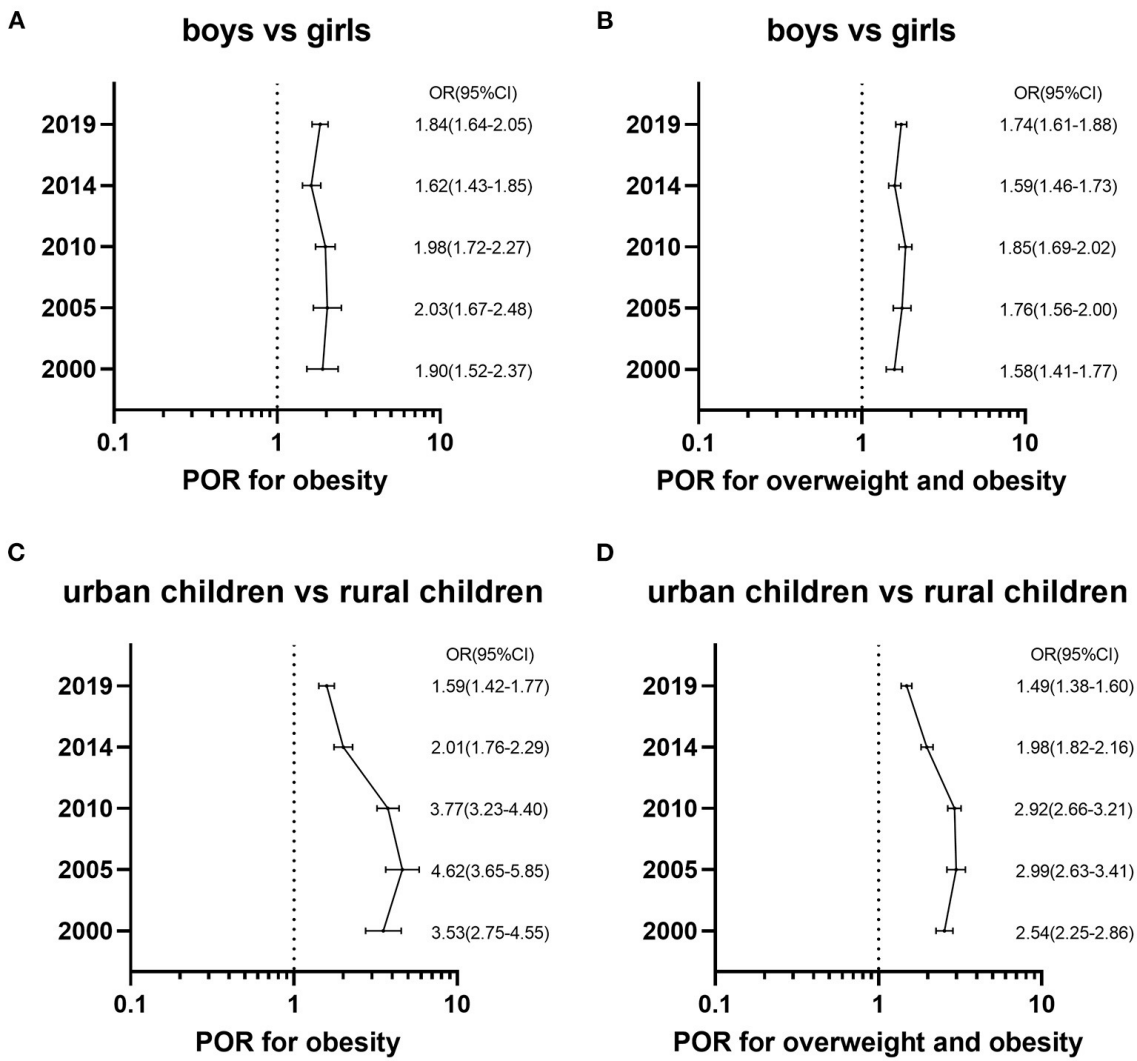
The prevalence odds ratios (POR) and the 95% confidence intervals (CI) for overweight and obesity of boys vs. girls with adjustment for the region, location and age in different survey years and urban children vs. rural children with adjustment for the region, sex and age in different survey years. **(A)** Shows PORs for obesity of sex disparity by using WGOC standard. **(B)** Shows PORs for overweight and obesity combined of sex disparity by using WGOC standard. **(C)** Shows PORs for obesity of urban-rural disparity by using WGOC standard. **(D)** Shows PORs for overweight and obesity combined of urban-rural disparity by using WGOC standard.

The PORs for obesity and the combined overweight and obesity of boys compared to girls in different survey years were estimated (Figure 3). Obesity prevalence was higher in boys than girls at each time point. The POR for obesity increased from 1.90 (95%CI: 1.52–2.37) in 2000 to 2.03 (95%CI: 1.67–2.48) in 2005 and from 1.62 (95%CI: 1.43–1.85) in 2014 to 1.84 (95%CI: 1.64–2.05) in 2019. However, the POR for obesity decreased from 2005 to 2014. The trends of the PORs for the combined overweight and obesity presented a similar pattern.

The PORs for overweight and obesity of urban children compared to rural ones from 2000 to 2019 were calculated (Figure 3). Obesity prevalence in rural areas was remarkably lower than urban areas in each survey year. However, the PORs for obesity had decreased since 2005, and the POR declined from 4.62 (95%CI: 3.65–5.85) in 2005 to 1.59 (95%CI: 1.42–1.77) in 2019. The trends of the PORs for the combined overweight and obesity showed an identical pattern.

## Discussion

In this study, we observed that the childhood prevalence of obesity and that of overweight and obesity combined in Henan province of China increased significantly from 2000 to 2019 based on WGOC standard. After stratified by age, the prevalence increased significantly, especially among participants in the 10 to 12 years. After stratified by location and sex, the prevalence also increased significantly, especially among the boys. To our knowledge, this is the first study to analyze secular trends in overweight and obesity prevalence among children in Henan province of China from 2000 to 2019. Our findings were also in agreement with accumulating evidence, which showed that obesity prevalence among Chinese students aged 7–18 years changed from 0.1 to 5.0% between 1985 and 2010, and that the overweight and obesity prevalence in Chinese students aged 7–12 years changed from 17.1 in 2010 to 22.5% in 2014 (1, 17). Several studies used other standards to distinguish overweight and obesity have also observed that there was an increasing trend in obesity prevalence of Chinese children (23–28). What's more, the trends of obesity in the present study were also consistent with previous studies in other countries (29–33).

Studies have revealed the role of hereditary, environmental and socioeconomic factors in the susceptibility and development of overweight and obesity (34). Given that genes may not change during a short time, the dramatic increase in the prevalence of overweight and obesity among children in Henan province of China from 2000 to 2019 was mostly driven by environmental and social factor (28). The output of grain in Henan province increased significantly over the past 20 years, so the adequate and abundant food supply may have provided a basis for the epidemic of childhood overweight and obesity (26). The increase of per capita gross domestic product (GDP), urbanization, and the decrease of Engel coefficient (a low Engel's coefficient reflects a higher living standard) in Henan province reflected the rapid economic growth, which led to westernized dietary pattern (25). This alteration may partly account for the trends of childhood overweight and obesity (24, 35). What's more, unhealthy lifestyles such as an increase in sedentary behavior, less physical activity, and shortened sleep duration were related to childhood overweight and obesity in China (34, 36, 37).

We also observed that there was a gender difference in obesity/overweight and obesity combined prevalence, and the gender difference have changed over time. The prevalence of obesity/overweight and obesity combined in boys were higher than those of girls during the past 20 years in our study. The PORs of obesity/overweight and obesity associated with sex in our study showed rising trend from 2000 to 2005 and from 2014 to 2019, but showed a decreasing trend from 2005 to 2014. Previous study also observed that the rate of obesity was higher in boys and the increasing pace in boys was faster than girls among Chinese students from 2000 to 2005 (29). Another study found that the increase of overweight was also more rapid in boys from 2000 to 2014 (23). Perception of childhood overweight and obesity could partly contribute to the sex disparity. In China, overweight and obesity in children are regarded as health and well being, especially in boys (26). On the other hand, Chinese girls are more likely to have a slender figure, and they are more willing to control body weight compared to their male counterparts (24, 26, 37). Also, the difference in dietary habits, physical activity levels and other lifestyles may play significant roles in sex differences of childhood overweight and obesity (23, 26). Besides, the sex differences in the susceptibility to obesogenic environments may also have a crucial role (29).

Besides, our results showed that the participants in urban areas presented higher obesity/overweight and obesity combined rates than those in rural areas. Previous study also found that overweight was a great problem in urban areas (23). Children and adolescents in urban areas have easy access to foods with high energy density and may take less exercise (17, 25, 26, 28). Besides, Engel's coefficient has been higher in rural than urban areas in Henan province from 2000 to 2019. Engle's coefficient means the ratio of household income spent on food and reflects the living standard of people (1, 17). Engel's coefficient with higher values reflects a poorer living standard (23). These changes may explain why we observed that the prevalence of obesity/overweight and obesity combined were both greater in children from urban areas than rural areas in Henan province.

In our study, the alteration of urban-rural differences of childhood obesity over the past 20 years can be classified as two stages. First, from 2000 to 2005, obesity prevalence was higher in urban children than in rural children, and the epidemic of obesity in urban areas was much faster than that in rural ones. Second, from 2005 to 2019, the urban-rural differences of obesity were getting narrower, although the prevalence of obesity was also greater in urban areas than rural ones. The change of urban-rural differences of childhood overweight and obesity combined also showed the same pattern. Consistent with our research, previous studies found that the disparities in obesity between urban-rural areas increased from 2000 to 2005 and decreased from 2005 to 2010 in China (17, 23, 25).

Economic development, policy implementation and changes in dietary patterns might help explain the findings. The urban grain rationing systems were abolished and the diverse food supply improved in urban areas firstly in 1993 (17), which may provide a basis for the increase of urban-rural disparity from 2000 to 2005 in Henan province. We also propose several reasonable explanations for the narrowing urban-rural disparity in obesity from 2005 to 2019 in Henan province. First, in 2011, our government implemented the Nutrition Improvement Program for Rural Compulsory Education Students (38), which probably played an important role in this change (1, 23, 25). Second, the gaps of Engel coefficients between urban and rural locations narrowed since 2005 in Henan province. Engel coefficients were 34.24 and 45.41% in 2005 for urban and rural areas, respectively, but declined further to 25.26 and 26.24% in 2019, respectively (39, 40). Third, the pace of urbanization in Henan province is rapid (17, 25), and several studies found that urbanization was one of the most critical drivers of the increase in obesity. Besides, economic growth promotes mechanization in rural areas in Henan province, which may lead to the decline of physical activity levels (41). Finally, rural residents may have less access to public health services about weight management (24, 36).

The present study provided the latest trends in overweight and obesity prevalence and had the following strengths. First, data were acquired from the largest national survey of Chinese children. The sample size was very large so that we have an adequate sample size to analyze data among different subgroups. Second, rigorous quality control ran through the whole research process, such as trained surveyors, corrected instruments, etc. Furthermore, the calculation of BMI was based on height and weight that were repeatedly measured by professionals rather than self-report, which increased the authenticity and reliability of the data. Third, the screening of overweight and obesity was on the basis of WGOC standard, which was suitable for the Chinese school-aged children (20).

There were also some limitations of our study that should not be ignored. First, we only calculated the prevalence based on the WGOC standard to assess the trend, which limited comparability with other studies. Second, the data were collected among Han nationality children in Henan province. However, the Han population accounted for 98.84% of the entire population of Henan province, and the population in Henan province made up 7.04% of the whole population in China. Finally, data collection was implemented in schools, so some school-aged children who could not attend school were excluded from our surveys (23).

In summary, a dramatic increase was observed in the childhood overweight and obesity prevalence in Henan province of China from 2000 to 2019. The sex disparity in overweight and obesity prevalence still existed with higher prevalence in boys than girls. Besides, the epidemic of childhood overweight and obesity in rural regions was much faster and the urban-rural disparity narrowed from 2005 to 2019. Our results indicated that it is very urgent to develop and implement sex-specific and area-specific preventive guidelines and intervention strategies to control childhood overweight and obesity.

## Data availability statement

The original contributions presented in the study are included in the article/Supplementary material, further inquiries can be directed to the corresponding author.

## Ethics statement

The studies involving human participants were reviewed and approved by Zhengzhou University Life Science Ethics Committee (ZZUIRB2021-56). Written informed consent to participate in this study was provided by the participants' legal guardian/next of kin.

## Author contributions

XW participated in the study design, data collection, and reviewed the manuscript. YZ, HL, and YH completed the data analyses, interpreted the data, and drafted the manuscript. RW, XW, CW, and RL took part in the study design and data collection. CH, GG, and XL took part in the study design and supervised field investigation. All authors made significant contributions to the article.

## Funding

This work was supported by the National Natural Science Foundation of China (Grant No. 82003478).

## Conflict of interest

The authors declare that the research was conducted in the absence of any commercial or financial relationships that could be construed as a potential conflict of interest.

## Publisher's note

All claims expressed in this article are solely those of the authors and do not necessarily represent those of their affiliated organizations, or those of the publisher, the editors and the reviewers. Any product that may be evaluated in this article, or claim that may be made by its manufacturer, is not guaranteed or endorsed by the publisher.

## Abbreviations

BMI: body mass index
CI: confidence interval
CNSSCH: Chinese National Survey on Students' Constitution and Health
IOTF: International Obesity Task Force
POR: prevalence odds ratio
SD: standard deviation
WGOC: Working Group on Obesity in China
WHO: World Health Organization.
