# Supplementary material for: Trends of overweight and obesity prevalence in school-aged children among Henan Province from 2000 to 2019
Source: Front Public Health. 2022 Dec 5;10:1046026. doi: 10.3389/fpubh.2022.1046026 (PMC9760942; doi:10.3389/fpubh.2022.1046026)
Supplement: Supplementary file 1 [file Table_1.DOCX]

**Table S1 Trends in overweight and obesity prevalence among different age groups across survey years in Henan Province, China**

| Category | 7 to 9 y | 10 to 12 y | 13 to 15 y | 16 to 18 y | Total |
| --- | --- | --- | --- | --- | --- |
| Obesity |  |  |  |  |  |
| 2000 | 3.22  (2.65-3.80) | 2.56  (2.04-3.07) | 2.33  (1.84-2.83) | 1.86  (1.40-2.32) | 2.51  (2.25-2.77) |
| 2005 | 7.41  (6.30-8.51) | 7.18  (6.09-8.27) | 4.17  (3.32-5.01) | 2.96  (2.25-3.68) | 5.43  (4.95-5.91) |
| 2010 | 10.20  (9.21-11.19) | 9.03  (8.09-9.97) | 5.45  (4.71-6.19) | 2.25  (1.77-2.73) | 6.73  (6.32-7.14) |
| 2014 | 11.77  (10.72-12.82) | 10.82  (9.80-11.83) | 4.63  (3.94-5.32) | 3.13  (2.56-3.70) | 7.59  (7.15-8.02) |
| 2019 | 13.58  (12.48-14.69) | 14.66  (13.51-15.81) | 8.40  (7.49-9.31) | 5.56  (4.81-6.30) | 10.56  (10.06-11.06) |
| *P* trend test | <0.001 | <0.001 | <0.001 | <0.001 | <0.001 |
| Overweight and Obesity |  |  |  |  |  |
| 2000 | 9.09  (8.15-10.03) | 9.53  (8.57-10.49) | 10.53  (9.53-11.54) | 10.11  (9.08-11.14) | 9.81  (9.32-10.30) |
| 2005 | 16.16  (14.60-17.71) | 17.96  (16.34-19.58) | 13.70  (12.25-15.16) | 12.04  (10.67-13.42) | 14.97  (14.21-15.72) |
| 2010 | 21.31  (19.97-22.65) | 21.62  (20.27-22.96) | 15.74  (14.54-16.93) | 11.25  (10.22-12.28) | 17.48  (16.86-18.10) |
| 2014 | 25.03  (23.62-26.45) | 25.60  (24.17-27.02) | 17.94  (16.69-19.20) | 14.72  (13.56-15.87) | 20.82  (20.16-21.48) |
| 2019 | 27.25  (25.81-28.69) | 30.71  (29.20-32.21) | 23.28  (21.89-24.66) | 22.28  (20.93-23.64) | 25.88  (25.17-26.60) |
| *P* trend test | <0.001 | <0.001 | <0.001 | <0.001 | <0.001 |

**Table S2 Trends in overweight and obesity prevalence among different genders and locations across survey years in Henan Province, China**

| Category | Urban boys | Urban girls | Rural boys | Rural girls | Total |
| --- | --- | --- | --- | --- | --- |
| Obesity |  |  |  |  |  |
| 2000 | 5.29  (4.56-6.03) | 2.40  (1.90-2.91) | 1.17  (0.81-1.53) | 1.12  (0.77-1.47) | 2.51  (2.25-2.77) |
| 2005 | 11.62  (10.27-12.97) | 5.93  (4.93-6.92) | 2.64  (1.96-3.31) | 1.53  (1.01-2.05) | 5.43  (4.95-5.91) |
| 2010 | 13.67  (12.55-14.80) | 7.11  (6.27-7.95) | 3.78  (3.16-4.40) | 2.36  (1.87-2.86) | 6.73  (6.32-7.14) |
| 2014 | 11.78  (10.72-12.83) | 7.98  (7.10-8.87) | 6.62  (5.81-7.43) | 3.97  (3.33-4.60) | 7.59  (7.15-8.02) |
| 2019 | 16.39  (15.20-17.59) | 8.98  (8.05-9.91) | 10.10  (9.11-11.09) | 6.67  (5.86-7.49) | 10.56  (10.06-11.06) |
| *P* trend test | <0.001 | <0.001 | <0.001 | <0.001 | <0.001 |
| Overweight and Obesity |  |  |  |  |  |
| 2000 | 16.95  (15.71-18.18) | 10.29  (9.29-11.29) | 6.49  (5.67-7.30) | 5.38  (4.64-6.13) | 9.81  (9.32-10.30) |
| 2005 | 26.81  (24.94-28.67) | 16.06  (14.52-17.61) | 9.95  (8.69-11.22) | 7.04  (5.96-8.12) | 14.97  (14.21-15.72) |
| 2010 | 31.02  (29.51-32.53) | 18.25  (16.99-19.51) | 12.20  (11.13-13.27) | 8.45  (7.54-9.36) | 17.48  (16.86-18.10) |
| 2014 | 30.83  (29.32-32.34) | 21.62  (20.28-22.97) | 18.14  (16.88-19.40) | 12.70  (11.62-13.79) | 20.82  (20.16-21.48) |
| 2019 | 36.17  (34.62-37.72) | 22.97  (21.61-24.34) | 25.72  (24.29-27.16) | 18.51  (17.24-19.77) | 25.88  (25.17-26.60) |

**Table S2 Continued**

| *P* trend test | <0.001 | <0.001 | <0.001 | <0.001 | <0.001 |
| --- | --- | --- | --- | --- | --- |
